# The complete chloroplast genome sequence of *Michelia macclurei* (Dandy, 1928) (Magnoliaceae), an important fire-resistant tree species

**DOI:** 10.1080/23802359.2022.2057251

**Published:** 2022-11-11

**Authors:** Shujing Wei, Sisheng Luo, Yingxia Zhong, Yufei Zhou, Zhao Song

**Affiliations:** Guangdong Academy of Forestry, Guangzhou, China

**Keywords:** *Michelia macclurei*, chloroplast genome, Magnoliaceae, phylogeny

## Abstract

*Michelia macclurei* (Dandy, 1928) is an evergreen broad-leaved tree species native to South China. This species has great ecological and economic importance. However, the genomic study of *M. macclurei* has lagged far behind. Here, we reported the complete chloroplast genome sequence of *M. macclurei*. The chloroplast genome size of *M. macclurei* was 160,139 bp, consisting of a pair of inverted repeat (IR) regions (26,575 bp), which was separated by a large single copy (LSC) region (88,167 bp) and a small single copy (SSC) region (18,822 bp). A total of 113 unique genes were annotated, including 79 protein-coding genes, 30 tRNA genes, and four rRNA genes. The overall GC content was 39.2%. Phylogenetic analysis based on 16 whole chloroplast genome sequences of *Michelia* species suggested that *M. macclurei* and *M. maudiae* are sister to each other, and jointly sister to *M. chapensis*.

*Michelia macclurei* (Dandy, 1928) belonging to the family Magnoliaceae, is an evergreen broad-leaved tree species endemic to South China (Jiang et al. 2017). The species is a valuable multipurpose tree that can be used for landscape and urban planting, soil improvement, catchment stabilization, and biological fire prevention forest belts (Wang et al. 2008; Niu et al. 2009; Zheng 2013). It can be planted in monoculture or a mixture with conifers (Xia et al. 2016). Despite its ecological and economic importance, the genomic study of *M. macclurei* has lagged far behind. Nowadays, chloroplast genome sequence has been extensively used for phylogenetic, comparative, and evolutionary studies, because of its lack of recombination, small effective population size, low rates of nucleotide substitutions, and usually uniparental inheritance (Lu et al. 2016; Firetti et al. 2017). Here, we reported the chloroplast genome sequence of *M. macclurei*, and reconstructed its phylogenetic relationship with other *Michelia* species.

The fresh leaves of *M. macclurei* were sampled from Guangdong Academy of Forestry, Guangdong Province, China (113.45°E; 23.20°N), and under special permission from Guangdong Academy of Forestry. A voucher specimen was deposited at Guangdong Academy of Forestry (www.sinogaf.cn, contact Shujing Wei, Weishujing2003@163.com) under the voucher number WSJ202107001. The DNA was extracted using DNA Plantzol Reagent (Invitrogen Trading (Shanghai) Co., Ltd, Shanghai, China). DNA library construction and 150-bp paired-end sequencing were performed on the Illumina HiSeq^4000^ platform. The chloroplast genome was assembled using GETORGANELLE pipeline (Jin et al. 2020), and annotated using Geneious Prime v.2021.1.1 (http://www.geneious.com) taking *Michelia chapensis* (MN897730) as a reference. The annotated chloroplast genome sequence of *M. macclurei* was deposited in GenBank (accession number: OK046128).

The chloroplast genome sequence of *M. macclurei* was 160,139 bp in length and exhibited the typical quadripartite structure, consisting of a pair of inverted repeat (IR) regions of 26,575 bp, separated by a large single copy (LSC) region of 88,167 bp and a small single copy (SSC) region of 18,822 bp. The chloroplast genome encoded a total of 131 genes, of which 113 (79 protein-coding genes, 30 tRNA genes, and 4 rRNA genes) were unique and 18 (7 protein-coding genes, 7 tRNA genes, and 4 rRNA genes) were duplicated in the IR region. A total of 16 genes were found to have introns, including 10 protein-coding genes and 6 tRNA genes. Of these genes, *clpP*, *trnA-UGC*, *trnI-GAU*, and *ycf3* had two introns, whereas *atpF*, *ndhA*, *ndhB*, *petB*, *rpl2*, *rpoC1*, *rps12*, *rps16*, *trnG-UCC*, *trnK-UUU*, *trnL-UAA*, and *trnV-UAC* had one intron. The overall GC content was 39.2%, whereas the CC contents in the IR, LSC, and SSC were 43.2%, 37.9%, 34.3%, respectively. Comparative chloroplast genome analyses of *M. macclurei* and other 15 previously reported chloroplast genomes of *Michelia* (Figure 1) showed the length of these chloroplast genomes ranged from 159,819 to 160,158 bp, the GC content ranged from 39.2% to 39.3%, and they collectively contained 113 common genes (e.g. Hinsinger and Strijk 2017; Wang et al. 2019; Deng et al. 2020; Sima et al. 2020; Zhai 2020; Zhou et al. 2020; Li et al. 2021). These results showed the chloroplast genomes within this genus are conserved in terms of genome size, genome structure, and gene content.

**Figure 1.**
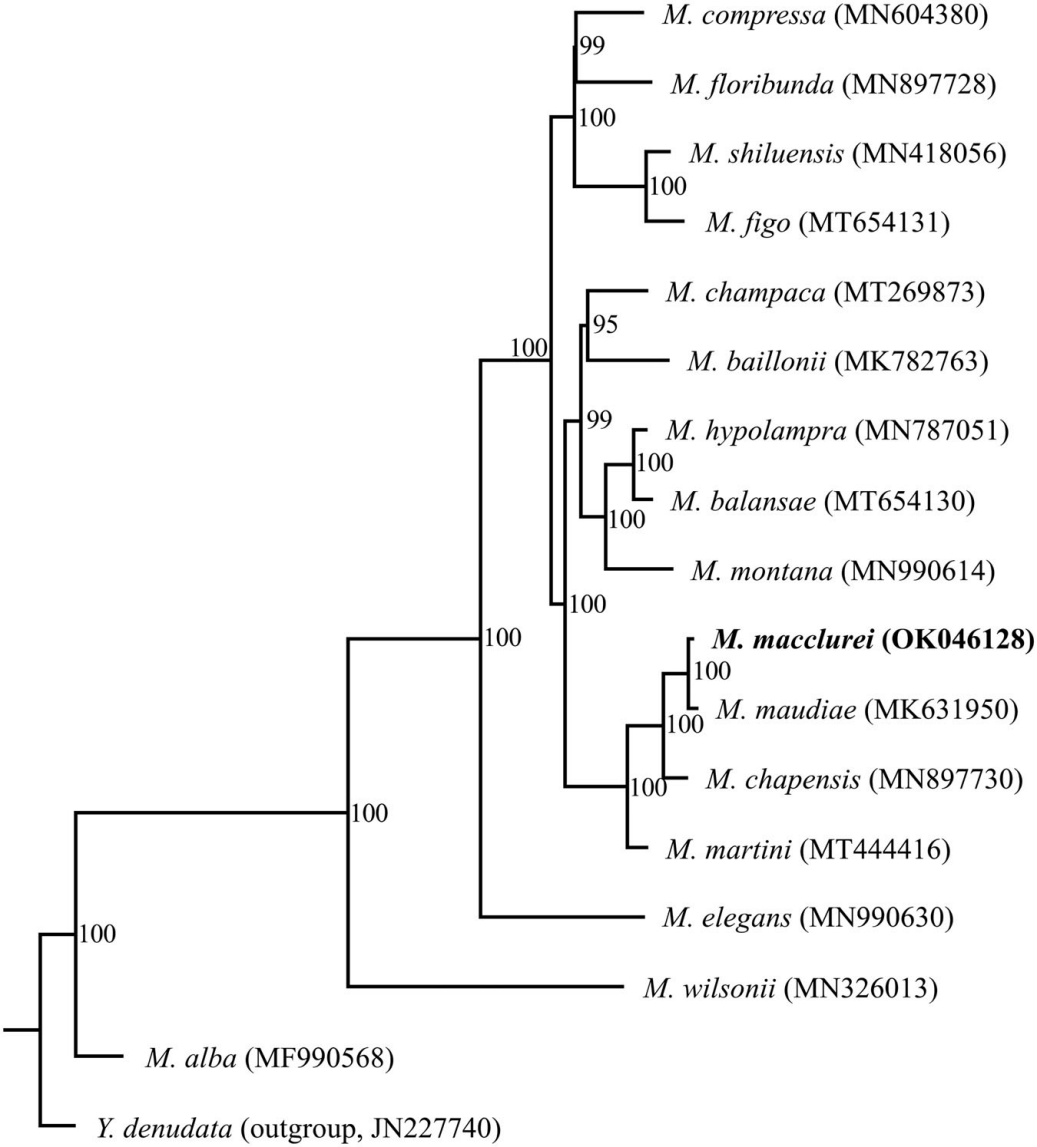
Phylogenetic tree inferred by maximum likelihood (ML) method based on complete chloroplast genomes of 16 *Michelia* species with *Yulania denudata* as an outgroup. Numbers near the nodes represent ML bootstrap values. The phylogenetic tree based on 79 protein-coding genes is completely consistent with this topology.

The phylogenetic relationship of *Michelia* was reconstructed using the maximum-likelihood (ML) method based on the multiple alignments of *M. macclurei* and other 15 previously reported chloroplast genomes of *Michelia*, with *Yulania denudata* (JN227740) as an outgroup. ML analysis was conducted based on two data sets: (1) the complete chloroplast genome sequences; and (2) a set of 79 common protein-coding genes, using RAxML-HPC v.8.2.8 (Stamatakis 2014) with 1000 bootstrap replicates on the CIPRES Science Gateway website (https://www.phylo.org/). The phylogenetic topologies based on these two data sets were completely consistent, with 100% bootstrap values at almost all nodes, and identically supported that *M. macclurei* and *M. maudiae* are sisters to each other, and jointly sister to *M. chapensis* (Figure 1).

## Data Availability

The genome sequence data that support the findings of this study are openly available in GenBank of NCBI at https://www.ncbi.nlm.nih.gov under the accession no. OK046128. The associated BioProject, SRA, and Bio-Sample numbers are PRJNA776621, SRS10788103, and SAMN22811516, respectively.
